# Pulmonary arterial hypertension associated with congenital heart disease: An omics study

**DOI:** 10.3389/fcvm.2023.1037357

**Published:** 2023-03-10

**Authors:** Maolin Zhao, Jian Liu, Mei Xin, Ke Yang, Honghao Huang, Wenxin Zhang, Jinbao Zhang, Siyi He

**Affiliations:** Department of Cardiovascular Surgery, Affiliated Hospital of Southwest Jiaotong University, General Hospital of Western Theater Command, Chengdu, China

**Keywords:** pulmonary arterial hypertension, congenital heart disease, omics, molecular mechanism, biomarkers

## Abstract

Pulmonary arterial hypertension associated with congenital heart disease (PAH-CHD) is a severely progressive condition with uncertain physiological course. Hence, it has become increasingly relevant to clarify the specific mechanisms of molecular modification, which is crucial to identify more treatment strategies. With the rapid development of high-throughput sequencing, omics technology gives access to massive experimental data and advanced techniques for systems biology, permitting comprehensive assessment of disease occurrence and progression. In recent years, significant progress has been made in the study of PAH-CHD and omics. To provide a comprehensive description and promote further in-depth investigation of PAH-CHD, this review attempts to summarize the latest developments in genomics, transcriptomics, epigenomics, proteomics, metabolomics, and multi-omics integration.

## 1. Introduction

Pulmonary hypertension (PH) is classified into five clinical subtypes: pulmonary arterial hypertension (PAH), PH associated with left heart disease, PH associated with lung diseases and/or hypoxia, PH associated with pulmonary artery obstructions, and PH with unclear and/or multifactorial mechanisms (1). PAH is a rare vascular disease with a morbidity of about 0.0001%–0.0002% (2) and is characterized by progressive development of pulmonary vascular remodeling, elevated pulmonary artery pressure, and heart failure (3, 4). In 2022, the European Society of Cardiology (ESC) and the European Respiratory Society (ERS) proposed new criteria for PAH as mean pulmonary artery pressure (mPAP) > 20 mmHg at rest, pulmonary arterial wedge pressure (PAWP) ≤ 15 mm Hg, and pulmonary vascular resistance (PVR) > 2 Wood units (1).

Congenital heart disease (CHD) is a structural cardiovascular malformation that occurs at birth. The overall prevalence of CHD was 8.98 per 1,000 live births in China (5). A systematic review and meta-analysis of the global birth prevalence of CHD showed that the birth prevalence of CHD increased year by year, reaching a maximum of 9.410‰ from 2010 to 2017. Notably, the prevalence of CHD in Asia was higher than in Europe and Americas for the first time, possibly because Asians have a higher genetic or environmental susceptibility to CHD (6). CHD is a common cause of PAH (7), but the prevalence of PAH in adults with CHD remains uncertain. A study from the Netherlands showed that the prevalence of PAH in adult CHD was 3.2% (8). However, PAH was unexpectedly common in adults from the European Congenital Heart Disease Survey Database, with a prevalence of PAH associated with CHD (PAH-CHD) of 28% (9). The 2012 National Audit of Pulmonary Hypertension in the UK reported that PAH-CHD patients accounted for 30.2% of all PAH patients (10). In addition, an epidemiological study of an insured pediatric population in the United States showed that approximately 75% children with PAH had a CHD diagnosis (11). Structural defects of the heart may increase cardiac load, pulmonary blood flow, and PAP, resulting in the development of PAH that further increases blood flow to the heart and raises the cardiac load, adversely impacting the patients’ quality of life, even leading to heart failure or death (12). PAH-CHD is an important type of PAH that belongs to the first main group and PH associated with complex CHD belongs to the fifth group (13), which is beyond the scope of this review's discussion. The clinical classification of PAH-CHD includes Eisenmenger's syndrome, PAH associated with prevalent systemic-to-pulmonary shunts, PAH with small/coincidental defects, and PAH after defect correction (14). PAH-CHD can induce progressive proliferation and migration of pulmonary vascular smooth muscle cells as well as permanent pulmonary vascular remodeling (15). Although there is a decreasing trend in the mortality of PAH-CHD, the symptoms of PAH-CHD can still worsen during a person's lifetime, and PAH is incurable (16). Although significant progress has been made for patients with Eisenmenger syndrome and therapies have substantially improved functional capacity and increased life expectancy, the long­term survival remains poor (17). Moreover, recent data showed that the presence of postoperative PAH is steadily being associated with increased mortality (18). If diagnosed and treated early, PAH-CHD can be completely reversed (19). Early diagnosis of PAH-CHD at the moderate or clinically subtle stage is critical and can enhance the cure rate of patients. However, the identification of biomarkers for PAH-CHD through conventional approaches for blood-based biomarker discovery is still a clinical challenge (20).

As a method to study diverse classes of biomolecules, omics is widely used to address biological systems in many dimensions (from genes to behaviors) and disclose the molecular properties underlying complex cellular behaviors (21), thus enabling individualized medicine at the molecular level (22). However, traditional single-omics studies are not comprehensive enough to better elucidate the biological processes at the systemic level, which require the integration of multi-omics data for a full analysis of biological systems (23). The pathogenesis of complex diseases such as PAH-CHD can be studied at different omics levels. Many of the methods currently used to assess the severity of PAH and evaluate treatment outcomes (e.g., right heart catheterization, cardiac function class, and 6-min walk distance) have significant limitations (15). Without early diagnosis and appropriate treatment, the survival rate of PAH-CHD is low. Therefore, there is an urgent need to identify valuable biomarkers to guide the diagnosis and treatment of PAH-CHD. This review summarizes recent multi-omics studies on PAH-CHD from the aspects of genomics, transcriptomics, epigenomics, proteomics, metabolomics, and the latest progress in multi-omics integration, which is helpful to understand the mechanism of PAH-CHD and guide diagnosis and treatment.

## 2. Genomics and PAH-CHD

Genomics is the study of an organism's total genetic contents including the genes, sequences, alignments, and structures; thus, it can provide an outlook for investigating biological problems starting with the most basic DNA code of life (24). Genome sequencing technologies are currently widely employed in research laboratories due to the rise of high-throughput sequencing technologies which are faster and have a greater output-to-cost ratio (25).

### 2.1. *SOX17* in PAH-CHD

SRY-related high-mobility-group (HMG) box transcription factor 17 (*SOX17*) is a novel risk gene of PAH (26). Numerous studies have shown that PAH caused by pathogenic variants of *SOX17* is frequently associated with CHD, hemoptysis, and radiological abnormalities. Zhu et al. (27) reported that *SOX17*, together with other PAH-CHD–associated genes including *TBX4*, *LGD*, *D-Mis*, *NOTCH1*, *PTPN11*, and *PSMD12* played an important role in PAH-CHD. Montani et al. (28) followed-up 20 patients with PAH carrying *SOX17*, seven of them were PAH-CHD (35%). Furthermore, Zhu et al. performed whole-exome sequencing in 256 patients with PAH-CHD and showed that *SOX17* had rare variants associated with PAH-CHD, which was highly restricted during development and encoded a transcription factor involved in Wnt/β-linked protein and Notch signaling (29). *SOX17* was verified to be a risk gene for PAH-CHD in PAH children with important factors of bone morphogenetic protein receptor type 2 (*BMPR2*), *TBX4*, and *SOX17* (30).

### 2.2. *BMPR2* in PAH-CHD

Mutations in the *BMPR2* gene, a member of the transforming growth factor beta (TGFβ) receptor superfamily, significantly increase the risk of developing hereditary PAH (31) and have been identified as a major genetic cause of PAH (30, 32, 33). However, it is controversial whether *BMPR2* is a risk gene for PAH-CHD. Roberts et al. (34) detected *BMPR2* mutations in 6% of a mixed cohort of adults and children with PAH-CHD. Similarly, another study found significant downregulation of *BMPR2* in the plasma of PAH-CHD patients and hypoxia-induced pulmonary artery smooth muscle cells (PASMCs) (35). However, a recent study found only 7 (2.7%) patients carrying rare deleterious *BMPR2* variants in a cohort of 258 patients with PAH-CHD and concluded that *BMPR2* variants were not a common cause of PAH-CHD (36).

### 2.3. Genetic variants in pediatric patients with PAH-CHD

Endothelin-1 (ET-1) is important in children with severe PAH-CHD, and previous studies have found a variant with minor allele adenine insertion in the 5′-untranslated region (5′-UTR) of the *EDN1* gene of PAH-CHD (37). Array comparative genomic hybridization (CGH) is a powerful tool for identifying and characterizing complex genomic rearrangements of less than 5–10 megabases (Mb), enabling a 10%–20% increase in the detection of non-equilibrium cryptic rearrangements, such as deletions and/or duplications. Dell’edera et al. (38) used array CGH, karyotyping, and molecular cytogenetics for genomic analysis to evaluate a case of multi-organ dysfunction (malformed neonate, complex CHD, PAH) and found that the 7q35q36.3 deletion and the accompanying 20q13.2q13.33 duplication resulted in *SHH*, *KCNH2*, *PRKAG2*, and *KMT2C* deletions and *GATA5*, *CHRNA4* and *GNAS* duplications in the affected child. A recent study suggested that the genes rs1799983, rs2070744, and rs61722009 encoding endothelial nitric oxide synthase (eNOS) may be risk factors for neonatal PAH-CHD patients in South Fujian (39).

## 3. Transcriptomics and PAH-CHD

Detection of genes expressed in specific physiological and pathological states through transcriptomics and next-generation sequencing provides detailed insights into cellular phenotypes (40). Transcriptomic studies of PAH-CHD involve the analysis of the expression of all types of RNA transcripts (e.g., messenger RNA [mRNA], non-coding RNA [ncRNA], microRNA [miRNA]) in a given cell or tissue type. In contrast to the genome which is essentially static, the transcriptome changes over time in response to cellular, environmental, and developmental stimuli.

### 3.1. Studies of miRNA associated with PAH-CHD

Micro RNAs (miRNAs) are a class of small endogenous ncRNAs that are important regulators of many genes; they act by interacting with the 3′-UTR of specific mRNA targets, leading to translation repression or transcript degradation. Many miRNAs have been reported to play a critical role in the development and progression of PAH (41–43) and CHD (44), highlighting their potential use as PAH biomarkers and therapeutic tools. Therefore, miRNAs may be used as early diagnostic and prognostic markers for PAH-CHD. miR-19a expression was enhanced in the blood of PAH-CHD patients compared to CHD patients and has been shown to be a significant marker of PAH-CHD (20). Long et al. (42) collected serum from 61 patients with PAH-CHD and 53 patients with CHD and found that serum miR-27b was upregulated in patients with PAH-CHD, while miR-451 was downregulated in patients with PAH-CHD, miR-27b and miR-451 were associated with B-type natriuretic peptide (BNP) and asymmetric dimethylarginine (ADMA), and that the associations were significantly correlated with disease severity. Similarly, recent studies have also found that the expression of miR-204 and miR-451 was significantly reduced in the blood of children with PAH-CHD compared with CHD and the healthy control group, suggesting that miR-204 and miR-451 could be used as diagnostic biomarkers for PAH-CHD and that combined detection of miR-204 and miR-451 was more valuable for the diagnosis of CHD-PAH (45). Another study also showed that the level of plasma miR-204 in children with PH-CHD was lower than in children with CHD, and miR-204 expression may be one of the indicators to judge the severity of PH and monitor response to PH therapy (46). In addition, a previous article found that the expression of circulating miR-21 of PAH-CHD patients was higher than that of normal controls. However, patients with heart failure had significantly lower expression of circulating miR-21 and left ventricular dysfunction (47). miR-223 is a potential circulating biomarker and small molecule drug for the diagnosis and treatment of PAH. Zeng et al. (48) found that female patients with PAH-CHD had lower serum miR-223 levels than healthy patients. It was also seen for the first time that miR-223 could regulate PASMC proliferation, migration, and actomyosin reorganization through its new targets RhoB and myosin light chain of myosin II (MLC2), thereby leading to vascular remodeling and PAH. MiR-98 shows lower expression in PAH-CHD patients than in CHD patients and can be used as a diagnostic marker of PAH-CHD (49). The expression of miR-509-3P in the serum of PAH-CHD patients was lower than that of the normal group, and the diagnostic value of circulating serum miR-509-3P for PAH is similar to that of echocardiography. Furthermore, the combination of MiR-509-3p and echocardiography further improves the diagnostic efficiency of PAH (50). Ma et al. (51) found that miR-27B was up-regulated in PAH-CHD patients compared to CHD patients, and the expression level of miR-27B was positively correlated with preoperative mPAP.

### 3.2. Studies of circular RNAs associated with PAH-CHD

NcRNAs play an important role in the pathogenesis of PAH (52). Circular RNAs (circRNAs) are a new type of ncRNA that play an important role in regulating cellular metabolism by binding to target miRNAs or by direct interacting with proteins (53, 54). They have become a hotspot of ncRNA. circRNAs are covalently closed RNA molecules produced by reverse splicing process, and their unique circular structure in absent 5′ caps or 3′ polyadenosine tail can protect them from degradation by RNA exonucleases, making them stable and abundant in tissues and body fluids, particularly in eukaryotes (55). This feature of circRNAs makes them promising clinical biomarkers for PAH-CHD. One study reported that circRNA hsa_circ_0003416 was significantly downregulated in the plasma of PAH-CHD patients compared to the CHD and control group, and negatively correlated with BNP (56). Using a bioinformatics approach, Su et al. (57) identified 27 differentially expressed circRNAs (3 up-regulated and 24 down-regulated), including downregulatedcirc_003416 and upregulated circ_005372 in PAH-CHD compared with CHD.

## 4. Epigenomics and PAH-CHD

Epigenomics is an omics approach to alter gene expression without changing the DNA sequence, and emerging epigenetic tools can be used for prevention, diagnosis, and therapeutic markers. Epigenetic mechanisms include DNA methylation and histone modifications (58). High-throughput genomic technologies based on next-generation sequencing now allow precise quantification and analysis of RNAs and species, allowing genome-wide studies of epigenetic or regulatory mechanisms, including deoxyribonucleic acid methylation, histone methylation, acetylation, and transcription factor binding (59). Epigenomic studies have played an indispensable role in revealing disease-associated epigenetic markers and are now applied to the study of various cancers (60), lung diseases, and cardiovascular diseases (61). Currently, there are relatively few reports on the epigenomic aspects of PAH, especially the epigenomic study of PAH-CHD has not been reported. Meloche et al. (62) revealed, for the first time, the critical role of the epigenetic reader Bromodomain Containing Protein-4 (BRD4) in the physiopathology of PAH by demonstrating that BRD4 acts as a coactivator in PAH to promote the transcription of genes leading to cell cycle progression. Another study found that increased BRD4 expression in the coronary arteries of PAH patients contributed to vascular remodeling and development of co-morbiditie (63). Chen et al. (64) reported that epigenetic upregulation of mitochondrial dynamic protein-49 (MiD49) and MiD51 increased mitosis, which drove pathological proliferation and resistance to apoptosis to promote PAH.

## 5. Metabolomics and PAH-CHD

Metabolomics characterizes the metabolites present in a sample or matrix, including the amino acids, fatty acids, carbohydrates, and other compounds produced by metabolic processes in biological fluids, cells, and tissues. It has traditionally been used to identify biomarkers for diagnosis and prediction of disease (65). With better understanding of pulmonary vascular disease, metabolic abnormalities have been identified as an important factor of PAH development and progression (66).

During pulmonary vascular stenosis, occlusion, and right heart failure, the metabolic profile in tissues and blood changes significantly, making it feasible to characterize the metabolic disturbances underlying the disease, discover biomarkers for risk stratification or prognosis, and identify new therapeutic targets. PAH-CHD patients had significantly higher plasma concentration of homocysteine (Hcy) and ADMA than CHD as well as healthy controls. Furthermore, Hcy levels were higher in the cyanotic PAH-CHD patients than the acyanotic patients in the same group (67). The concentration of Hcy increased with a significantly negative correlation in children with PH-CHD and is a potential biomarker to predict PH (68).

Iron is an essential trace element that plays a key role in normal physiological processes, and the occurrence of PAH is also associated with iron deficiency (ID) (69). For example, ID was found to be highly prevalent in patients with PAH-CHD, with 39% of 153 PAH patients suffering from ID (70). Recently, He et al. (71) performed a metabolomics study of the plasma of patients with PAH-CHD, wherein a total of 193 different metabolites were measured at different time points of defect repair: prior to cardiopulmonary bypass (CPB) after anesthesia (Pre), immediately after CPB (T0), 24 h (T24), and 48 h (T48) after defect repair. Alterations in 17 metabolites were significantly associated with a decrease of mPAP at T48: propionylcarnitine, butenylcarnitine, isobutyryl-L-carni-tine, hexanoylcarnitine, PC[16:0/22:4(7Z, 10Z, 13Z, 16Z)], 7-methyl-guanine, bilirubin, 3-amino-2-oxazolidone, isoleukylproline, anserine, L-homoserine, N4-acetylsulfamethoxazole, galactinol dihydrate, and daidzein 4′-O-glucuronide were positively correlated with mPAP decrease; PC [14:0/22:5(4Z, 7Z, 10Z, 13Z, 16Z)], hydroxyphenylacetylglycine, and guanosine monophosphate were negatively correlated with mPAP decrease. The gradients of blood gas indicators (DPAP, aHCO3, svcHCO3, and aPCO2) were positively correlated with the gradient of mPAP at T48 and were correlated with the changes of the shunt correction-related metabolites: propionylcarnitine, butenylcarnitine, isobutyryl-L-carnitine, and hexylcarnitine.

## 6. Proteomics and PAH-CHD

Proteomics is a discipline developed independently on genomics for characterizing the expression levels, post-translational modifications, protein-protein interactions, and other features of all proteins expressed in a complete organism (or cell) at a specific time and in a specific space, aiming to describe them as precisely as possible. Proteomics can also characterize the entire set of proteins and their isoforms in a cell, tissue, or biofluid to gain, at the protein level, a comprehensive understanding of the processes related to organism physiology and pathology (72). Complex processes such as transcriptional regulation, selective splicing, RNA editing, and protein execution occur during gene expression, thereby making measurements at the protein level more suitable for detecting clinical phenotypes than raw transcriptome data. Indeed, with the development of high-throughput mass spectrometry (MS) and analytical software, proteomics has become an important complement to genomic approaches (73). Proteomics technology used in the field of PAH-CHD can provide new specific markers for early diagnosis, while simultaneously detecting and monitoring the disease progression and prognosis, to provide new clues for the pathogenesis of PAH-CHD. Therefore, the study of PAH-CHD proteomics has irreplaceable clinical significance and application prospects.

### 6.1. Endothelin-1 in PAH-CHD

Endothelin is a peptide isolated from vascular endothelial cells having vasoconstrictive activity and can induce proliferation of vascular smooth muscle cells. Endothelin-1 (ET-1) plays an important role in the pathogenesis of PAH, and the ET-1 pathway is an important target for PAH-specific drug therapy (37). As early as 1991, Yoshibayashi et al. (74) found elevated endothelin concentrations in patients with PAH-CHD compared to patients without PH. Later, Huang et al. (75) observed increased expression of ET-1 and ET-1 receptors (ETRs), and activation of urvivin-Akt, urvivin-ERK1/2, and phos-pho-mTOR pathways in the pulmonary arteries and small arteries of PAH-CHD patients. Li et al. (76) found that circulating endothelial cells (CECs) and ET-1 levels were significantly elevated in patients with PAH-CHD than healthy controls, as well as positively correlated with the indicators for assessing the severity of PAH: mPAP, arterial partial pressure of oxygen, and arterial oxygen saturation. A study on 31 patients with PAH-CHD showed a significant positive linear correlation among mPAP and plasma ET-1, ADMA, BNP, and uric acid (UA) levels in all patients, and suggested that ET-1 may be a biomarker of PAH-CHD and can be used to pre-evaluate the effect of iloprost on PAH-CHD (15).

### 6.2. ADMA and BNP in PAH-CHD

Several studies reported aberrant expression of ADMA and BNP in PAH-CHD. A systematic review included 1,113 patients with PAH-CHD who had higher levels of BNP, N terminal-pro-fragment (NT-proBNP), ADMA, and vascular endothelial growth factor (VEGF). The study found that the ADMA concentration was elevated in patients with PAH-CHD compared with patients with CHD alone, while the VEGF expression was significantly higher in patients with persistent PAH and CHD after correction of underlying heart disease than in those with PAH-CHD (77). Another study also reported significantly elevated BNP and ADMA in the serum of patients with PAH-CHD compared with the CHD and control groups (45). In addition, Fang et al. (78) found that plasma ADMA level was significantly elevated in patients with PAH-CHD and could be used as a biomarker for identifying PAH-CHD and assessing the response to sildenafil therapy in patients with coronary artery disease. Furthermore, the specificity and sensitivity were 82.8% and 90%, respectively, at the threshold value of 0.485 umol/L for measurement of plasma ADMA in patients with severe PAH. Eisenmenger's syndrome (ES) could also be identified by plasma ADMA, with a threshold value of 0.85 umol/L, specificity of 85.2%, and sensitivity of 64.3%.

### 6.3. Differential proteins in reversible and irreversible PAH-CHD

When PAH-CHD pressure is significantly elevated, there is no ideal metric to determine whether PAP improves after treatment in these patients. Using proteomics techniques, finding key differential proteins that are responsible for whether postoperative PAP is reversible in patients with PAH-CHD has the opportunity to provide a basis for the treatment of PAH-CHD. With proteomic analysis, Huang et al. (79) identified 85 up-regulated proteins and 75 down-regulated proteins in PAH-CHD compared with normal lung tissues, including cytoskeletal proteins and collagen chains, predominantly implicated in cell adhesion, extracellular matrix, cytoskeleton, immune response, and complement pathways. Among these proteins, Caveolin-1, filamin A expression, and cathepsin D were significantly upregulated, and glutathione-transferase MU1 (GSTM1) was significantly downregulated in the irreversible PAH-CHD group; the expression of Caveolin-1, filamin A, and cathepsin D were positively correlated with whereas the expression of GSTM1 was negatively correlated with pathological grade. Transgelin, a 22-kD protein of the calponin family, is exclusively and abundantly expressed in the cytoskeleton of visceral and vascular smooth muscle cells in adult animals. It was shown that transgelin was highly expressed in PASMC of small pulmonary arteries in PAH-CHD tissues compared to normal lung tissues and showed positive correlation with pathological grading, especially in the irreversible PAH group (80). A study of serum urviving found that preoperative serum urviving in rats with irreversible PAH-CHD was significantly higher than that in rats with reversible PAH-CHD and that there was also a significant correlation between serum urviving and BNP, preoperative PVR index, and postoperative mPAP (81).

### 6.4. Potential protein biomarkers in PAH-CHD

Angiotensin-converting enzyme 2 (ACE2) is a major component of the vasoprotective axis of the renin-angiotensin system (RAS). The serum level of ACE2 protein in PAH-CHD patients decreased significantly with the increase of mPAP, which is likely a marker of severity and prognosis in PAH-CHD patients (82). Prostaglandins are also involved in the development of PAH, and previous studies have reported overexpression of cyclo-oxygenase 2 (Cox-2) protein, a catalyzer of prostaglandin, in the blood of children with PAH-CHD (83). Zhang et al. (84) performed the first proteomic analysis of plasma from PAH-CHD patients using iTRAQ technology and showed that about 190 differential proteins were detected in different types of PAH-CHD patients. Ten differential proteins were identified when comparing CHD and PAH-CHD patients (SAA1 protein, complement Factor H-related Protein 2, anti-Factor VIIIscFv, Carbamoyl-phosphate synthetase I, APCS protein, von-Willebrand factor, BRF1 protein, glyceraldehyde-3-phosphate dehydrogenase, glycosylphosphatidylinositol phospholipase D, and intestinal lactoferrin receptor). High mobility group box protein 1 (HMGB1) is a ubiquitous nuclear protein that is constitutively expressed in most cells. It has been reported that the HMGB1 level was significantly elevated in patients with PAH-CHD compared to patients without PAH and healthy controls, as well as correlated significantly with PAP and PVR (85). Similarly, Li et al. (86) found that plasma growth differentiation factor 15 (GDF15) level was significantly elevated in children with PAH-CHD compared to those with CHD without PAH, and positively correlated with functional class, uric acid, NT-proBNP, and hemodynamic indices. Pim-1 (provirus integration site for Moloney murine leukemia virus) kinase, a calcium/calmodulin-dependent serine/threonine protein kinase, is a downstream regulator of STAT3 and is implicated in apoptosis, proliferation, differentiation, and tumor formation. As reported by Zhu et al. (87), Pim-1 was highly expressed in patients with PAH-CHD compared to healthy controls and patients with CHD without PAH, which had significant value for the diagnosis of PAH-CHD when the plasma Pim-1 concentration reached 16.80 ng/ml, and had a reference value for the diagnosis of severe PAH-CHD when the plasma Pim-1 concentration was 20.53 ng/ml. Meng et al. (88) found that osteopontin (OPN) expression was higher in PAH-CHD patients than in those with CHD but no PAH, and positively correlated with pulmonary hypertension status. Furthermore, OPN partially acted through anb3-integrin-Akt and anb3-integrin-ERK1/2 cascades to enhance the proliferation and migration of PASMC in rats, and played an important role in vascular remodeling of PAH. Connective tissue growth factor (CTGF), which functions as a downstream mediator of TGF-β signaling is also closely associated with pulmonary vascular remodeling and may be a promising diagnostic biomarker. It was found that plasma CTGF level was significantly higher in patients with PAH-CHD and in patients with cyanotic PAH-CHD than in patients without cyanotic PAH-CHD (89). Copeptin has been used as a surrogate biomarker for arginine pressor (AVP) secretion. Gaheen et al. (90) evaluated plasma copeptin levels in 25 children with PAH-CHD and found that elevated plasma copeptin level in children was a good predictor of severe PAH and poor prognosis. Plasma copeptin level has significantly positively correlation with mPAP, PVR, and pulmonary blood flow, and negatively correlated with right ventricular diastolic function. Recently, it has been shown that endothelial inhibitory hormone was elevated in pediatric PAH-CHD patients compared with healthy controls and controls with CHD, and associated with disease severity, disease improvement, and poorer survival in PAH-CHD (91).

## 7. Integrating omics in PAH-CHD

Cybermedicine uses computational biology tools to integrate multi-omics big data to potentially improve the diagnosis, prognosis, and treatment of complex diseases, and is now successfully applied to PH, coronary heart disease, diabetes, chronic lung disease, and developments in medicine (92). With the integration of multi-omics data, more biological processes, disease types, and key mechanisms for customized therapy will be identified (93).

Yuan et al. (94) integrated proteomics and metabolomics for the first time, followed by bioinformatics analysis, and found many differential proteins, metabolites, and key pathways in patients with ventricular septal defect (VSD)-PAH compared to VSD controls. The protein alterations included upregulation of DBH, ADIO, ANPEP, GP1BA, and TFR1 and downregulation of GNAS. The abnormal metabolites were increased 5-HT, taurine, creatine, sarcosine, 2-oxobutanoate, as well as decreased vanillylmandelic acid, 3,4-dihy-droxymandelate, 15-keto- prostaglandin F2α, fructose-6-phosphate (F-6-P), L-glutamine, dehydroascorbate, hydroxypyruvate, threonine, L-cystine, 1- aminocyclopropane-1-carboxylate. The key pathways involved cAMP signaling pathway, ECM-receptor interaction, AMPK signaling pathway, HIF-1 signaling pathway, PI3K -Akt signaling pathway, and nepicastat hydrochloride monohydrate. In addition, three predictors (based on plasma concentrations of DBH, ANPEP, and ADIO) were detected in patients with congenital ventricular septal defect PH. Ma et al. (95) performed 16 S rRNA sequencing and metabolomics analysis on bronchoalveolar lavage fluid of PAH associated with the congenital Left-to-Right shunts (LTRS), patients with LTRS but no PAH, and the healthy group. The integration of multi-omics showed that the pulmonary microbes and metabolites may be potentially effective biomarkers. Specifically, microbial composition analysis indicated that the Bacteroidetes phylum was less abundantly altered, while *Lactobacillus*, *Alicycliphilus*, and *Parapusillimonas* were significantly altered, which may contribute to PAH-LTRS in children. Moreover, metabolome proﬁling data showed that metabolites involved in Purine metabolism, Glycerophospholipid metabolism, Galactose metabolism, and Pyrimidine metabolism were signiﬁcantly disturbed in the PAH-LTRS cohort.

## 8. Conclusions and future directions

The development of PAH-CHD is complicated, involving multiple signaling pathways and molecular mechanisms, which remains to be further studied for screening serum biomarkers, combination of diagnostic protocols, and individualized precision therapeutic strategies. Recent application of omics such as genomics, epigenetics, transcriptomics, metabolomics and proteomics has improved the understanding of the pathogenesis of PAH-CHD. Particularly, proteomics has provided new perspectives for the diagnosis, treatment, and prognosis of PAH-CHD. However, little work has been done on PAH-CHD epigenomics and metabolomics yet, especially the former, which should be given more attention in future research.
